# Saikosaponin A attenuates neural injury caused by ischemia/reperfusion

**DOI:** 10.1515/tnsci-2020-0129

**Published:** 2020-07-02

**Authors:** Xinying Wang, Guofeng Yang

**Affiliations:** Department of Neurology, Hebei Medical University, No. 361, East Zhongshan Road, Shijiazhuang 050017, Hebei, China; Department of Neurology, Harrison International Peace Hospital, No.180, East Renmin Road, Hengshui 053000, Hebei, China; Department of Geriatrics, Second Hospital of Hebei Medical University, No. 215, West Heping Road, Shijiazhuang 050000, Hebei, China

**Keywords:** ischemia, neuroprotection, stroke, toll-like receptors, HMGB1

## Abstract

**Background:**

Inflammation is involved in cerebral ischemia/reperfusion (I/R)-induced neurological damage. Saikosaponin A (SSa), extracted from *Radix bupleuri*, has been reported to exert anti-inflammatory effects. This article aimed to investigate whether SSa could ameliorate neuroinflammation mediated by ischemic stroke and the underlying mechanism.

**Methods:**

Rat middle cerebral artery occlusion (MCAO) model was employed in this study, and the cognitive and motor functions were detected by behavioral tests. Inflammatory cytokines in the serum were detected by ELISA kits. The expression levels of Toll-like receptor 4 (TLR4), nuclear factor-kappa B (NF-κB), and high-mobility group box 1 (HMGB1) in the brain tissues were assayed with Western blot.

**Results:**

Our results showed that SSa pretreatment could significantly reduce brain damage, improve neurological function recovery, and decrease the water content of brain tissues when compared with the model group. SSa pretreatment significantly reduced the serum HMGB1 level and downregulated the contents of inflammatory cytokines including tumor necrosis factor-α, interleukin-1 beta, and interleukin-6. Furthermore, SSa pretreatment could attenuate the decreased TLR4 and nucleus NF-κB in the brain of MCAO rats. The protein level of HMGB1 in the nucleus was significantly upregulated in the SSa pretreatment group.

**Conclusion:**

Our results suggested that the pretreatment with SSa provided significant protection against cerebral I/R injury in rats via its anti-inflammation property by inhibiting the nucleus HMGB1 release.

## 1 Introduction

Cerebral ischemia/reperfusion (I/R), or cerebral ischemic injury, is a common refractory disease with potentially serious and long-term disability. It is one of the main causes of death all over the world [1]. In cerebral I/R, the blood supply to the brain is initially limited and subsequently recovered with blood flow oxygenation [2,3]. It results in brain tissue death, such as cerebral infarction, caused by insufficient blood and oxygen supply due to cerebral vascular occlusion. Cerebrovascular dysfunction induced by cerebral I/R is considered to be a very important cause of nervous system damage in ischemic stroke. Finding effective methods to prevent cerebral I/R injury is of great significance in the ischemic stroke treatment.

It is well known that the pathophysiology of ischemic stroke includes not only inflammatory components but also thrombotic components. Due to the impeded blood supply, pathophysiological events, such as oxidative stress, abnormal energy metabolism, abnormal ion balance, glutamate excitotoxicity, inflammation, and apoptosis, occur in the disease [4]. Leukocyte recruitment is reported to occur in the global and focal cerebral I/R models. Cerebral I/R triggers the inflammatory cascade and subsequently causes cytotoxic neuronal cell death, which is also called secondary brain injury [5,6]. The inflammatory response associated with brain damage is primarily dependent on Toll-like receptor 2 (TLR2) and Toll-like receptor 4 (TLR4) [7]. The endogenous TLR ligand, high-mobility group box 1 (HMGB1), is involved in the activation of inflammatory cytokine expression in infiltrating macrophages [8].

HMGB1 release triggers inflammatory processes to exacerbate neuronal damage in the post-ischemic brain during acute ischemic brain injury induced by *N*-methyl-d-aspartate [9,10]. Consistently, infarct formation is inhibited, and the blood–brain barrier (BBB) is protected by the administration of the HMGB1 MAb in the postischemic brain [11]. In addition, HMGB1 knockdown mediated by siRNA, as well as HMGB1 inhibition mediated by HMGB1 A-box, significantly reduces cerebral infarction volume in the rat middle cerebral artery occlusion (MCAO) model [12,13]. Mechanically, the release of HMGB1 leads to the activation of nuclear factor-kappa B (NF-κB), which usually induces increased pro-inflammatory cytokines, adhesion molecules, and chemokines and exacerbates cellular oxidative stress [14,15]. It is reasonable to reduce HMGB1 to inhibit NF-κB activation. Therefore, to reduce the extracellular HMGB1 level and subsequently inhibit reactive oxygen species, inflammatory necrotic cells and inflammatory cytokine activity remain important effective targets for brain I/R injury treatment.

Saikosaponin A (SSa), derived from *Radix bupleuri*, is widely used in Chinese traditional medicine and has anti-inflammatory effects. SSa suppresses inducible nitric oxide synthase (iNOS), cyclooxygenase-2 (COX-2), and proinflammatory cytokines by inhibiting NF-κB activation in carrageenan-induced paw edema, showing effective anti-inflammatory activity [16]. Furthermore, SSa has also been demonstrated with anti-inflammatory and antifibrotic actions on CCl4-induced liver damage [17]. Saikosaponin-d (SSd) alleviates CCl_4_-induced acute hepatocellular injury, possibly by inhibiting oxidative stress and NLRP3 inflammasome activation in the HL-7702 cell line [18]. Another study showed that SSd attenuated lipopolysaccharide-induced depressive-like behaviors via inhibiting microglia activation and neuroinflammation [19]. However, the neuroprotective effect of SSa in MCAO rats has not been studied yet. As far as we know, our research is the first to investigate the effects of SSa on cerebral I/R injury. Therefore, the aim of this article was to explore whether SSa could ameliorate ischemic stroke-induced neuroinflammation and brain damage, and whether the underlying mechanism was related to the HMGB-1/TLR4/NF-κB signaling pathway.

## 2 Materials and methods

### 2.1 Animal model and drug administration

**Figure 1:**
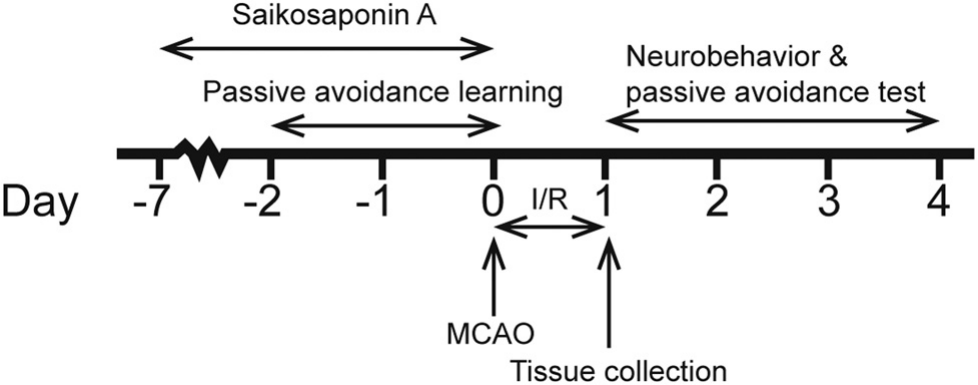
Experiment procedures.


Male Sprague-Dawley rats (200 ± 20 g), obtained from Shanghai Laboratory Animal Center (Shanghai, China), were used to build the MCAO model to imitate ischemic re-infusion condition. SSa was purchased from Xi’an Imaherb Biotech Co., Ltd (Xi’an, China), with 99% purity and batch no. 83-67-0. The SSa was dissolved in 0.9% physiological saline as a stock solution, stored at −20°C, and diluted with medium with a specific dosage for each group. The animals were allowed to get access to food freely. The experimental procedures are illustrated in Figure 1. The animals were divided into five groups including three doses of SSa groups, animals in sham and MCAO (model) groups with no SSa treatment. Each group has 14 animals. Different doses of SSa were added to the chow diet 1 week before MACO, which were 0.001% (S1 group), 0.01% (S10 group), and 0.1% (S100 group) weight/food weight ratio, respectively. Through the midabdominal line incision, the operation exposed the right common carotid artery, external carotid artery, and internal carotid artery (ICA). A 0.26 mm nylon filament coated with silicon resin was introduced into the right ICA and advanced about 18–20 mm to the starting point of the middle cerebral artery to occlude. MCAO was considered successful after observing the left hemiparesis. After 1 h of MCAO, reperfusion of blood for 24 h was established by carefully withdrawing the nylon filament. The sham rats received similar surgical procedures only without nylon filament on the ICA to block the blood flow. None of the rats in the Sham group died (0 of 14 rats). Mortality was 28.6% (4 of 14 rats) in the S0 and S1 group, 14.3% (2 of 14 rats) in the S10 group, and 21.4% (3 of 14 rats) in the S100 group. Thus, the number of animals used in the study was as follows: 7 rats for the Sham subgroup, 5 rats for S0 and S1 subgroup each, 6 rats for the S10 subgroup, and 5–6 rats for the S100 subgroup. The dead rats were excluded from further analysis.

**Ethical approval:** All experimental procedures were carried out in accordance with the People’s Republic of China Laboratory Animal Use and Care Law, and all experiments were approved by the Animal Care and Use Committee in the Second Hospital of Hebei Medical University.

### 2.2 Evaluation of motor function

Motor function was evaluated according to the Longa method by an examiner who was blinded to the experimental groups after the rats recovered from unconsciousness. The evaluation was scored as follows: 0 point, no symptoms of neurological deficits; 1 point, the contralateral forepaws could not be fully extended; 2 points, decreased resistance to lateral push; 3 points, unidirectional circling while walking; 4 points, could not walk by themselves; and 5 points, paralyzed. Higher scores indicated more severe neurological dysfunction.

### 2.3 Cognitive function examination

Rats show preference of darkness. The device was designed to have two rooms, one bright and the other dark. There was one door adjacent to the two rooms and a live metal mesh at the bottom of the darkroom. An animal would be subjected to a 0.5-mA electric shock when it entered the darkroom. Two days before the MCAO, the rats were placed in the bright room and turned to the darkroom. The time spent by the rat to enter the darkroom was measured and considered as an incubation period. The time spent in the dark chamber was recorded to assess learning and memory skills. If the rat did not enter the darkroom within 2 min, the learning exercise was completed. Longer step incubation periods or shorter darkroom times represented better cognitive function. The examination deadline was 5 min. The passive avoidance test was carried out blindly 4 days after MACO.

### 2.4 Measuring the cerebral infarct and edema

Tissues were collected 24 h after MCAO completed. After deep anesthesia with 10% chlorine hydrate, the rat brains were taken out and placed at −20°C for 15 min. After cutting into 2 mm thick slices on the ice platform, the brain slices were stained with 2% 2,3,5-triphenyltetrazolium chloride (TTC) at 37°C for 30 min and then fixed with 4% formaldehyde. The white area of cerebral infarction and the red non-ischemic area were measured using Image-Pro Plus using the formula: infarct volume% = ([reverse hemisphere volume − side hemisphere noninfarct area]/reverse hemisphere volume) × 100%. The brain was quantified with an electronic scale as wet weight and then dried to a constant weight in a desiccating oven at 105°C as dry weight. Total brain water content was calculated using the formula: ([wet weight − dry weight]/wet weight) × 100%.

### 2.5 Lactate dehydrogenase (LDH) activity detection

The ischemic brain tissues from each group were taken from the same area from bregma +0.7 to −4.3 mm after 24 h of MCAO. Detection of LDH activity was performed in the brain tissues using a commercial kit according to the manufacturer’s instructions (Nanjing Jiancheng Bioengineering Institute, China).

### 2.6 Measurement of inflammatory cytokines in serum

Commercial ELISA kits were used to detect serum inflammatory cytokine levels including interleukin-1 beta (IL-1β), interleukin-6 (IL-6), tumor necrosis factor-α (TNF-α), and HMGB1 following the standard manual (Nanjing Jiancheng Bioengineering Institute, China). After 24 h of MACO, 1 ml of blood samples obtained from the rat hearts was centrifuged at the speed of 2,000 × *g* for 10 min at 4°C, and then, the supernatants were used to detect inflammatory cytokines.

### 2.7 Western blot for TLR4, NF-κB, and HMGB1 expression

After behavioral testing, ischemic cortex tissue samples were collected for Western blot analysis. The total protein was lysed, and 20 μg protein samples were separated on 10% SDS polyacrylamide gels and then transferred to PVDF membranes. The membranes were blocked for 1 h in 5% skimmed dry milk buffer at room temperature and incubated overnight with the primary antibody (TLR4, NF-κB, and HMGB1; 1:1,000; Milliporesigma, USA). After being washed three times with TBST, the membranes were incubated for 2 h at room temperature with the secondary antibody conjugated with the spicy root peroxidase. The protein expression levels were detected by the electrochemical luminescence detection system and analyzed by the Image-J software.

### 2.8 Statistical analysis

Data were presented as mean ± SD. Multiple group comparisons were performed by one- or two-way analysis of variance (ANOVA), followed by Turkey’s test. *P* <0.05 was considered statistically significant.

## 3 Results

### 3.1 Effects of SSa on motor function of rats after I/R treatment

**Figure 2:**
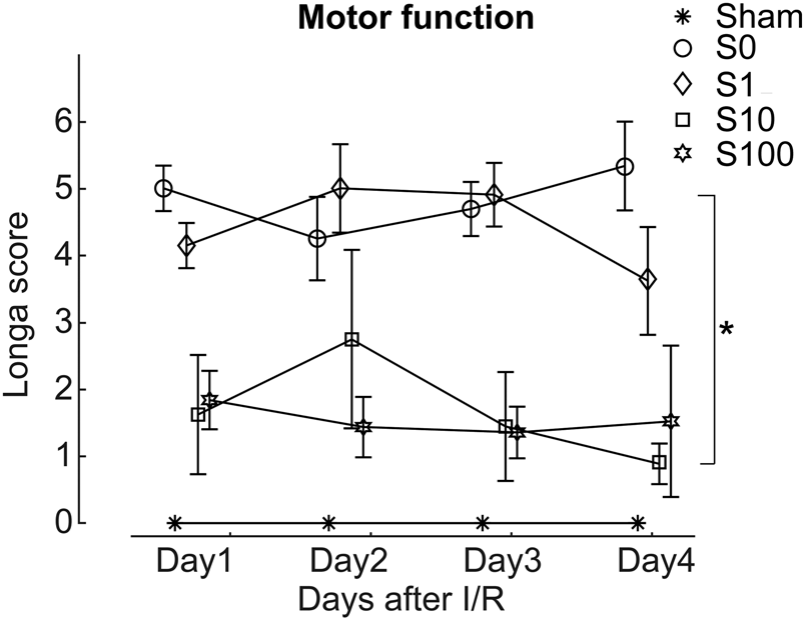
Saikosaponin A protected motor function induced by the I/R treatment. Animals delivered with 0.01% and 0.1% (group s10 and s100) showed better motor function when compared with the model group. However, 0.001% delivery of Saikosaponin A did not show protection effect. *p < 0.05, S10 compared with model; S100 compared with model.

The Longa score was used to assess motor function in rats (Figure 2). Rats in the sham group showed good baseline motor function. After MCAO, I/R group rats had lower neurobehavioral scores at different stages than the sham group. After SSa treatment, motor dysfunction was rescued, and neurobehavioral scores were higher than the I/R group, especially in the S10 and S100 groups.

### 3.2 Effects of SSa on the cognitive function of rats after I/R treatment

**Figure 3:**
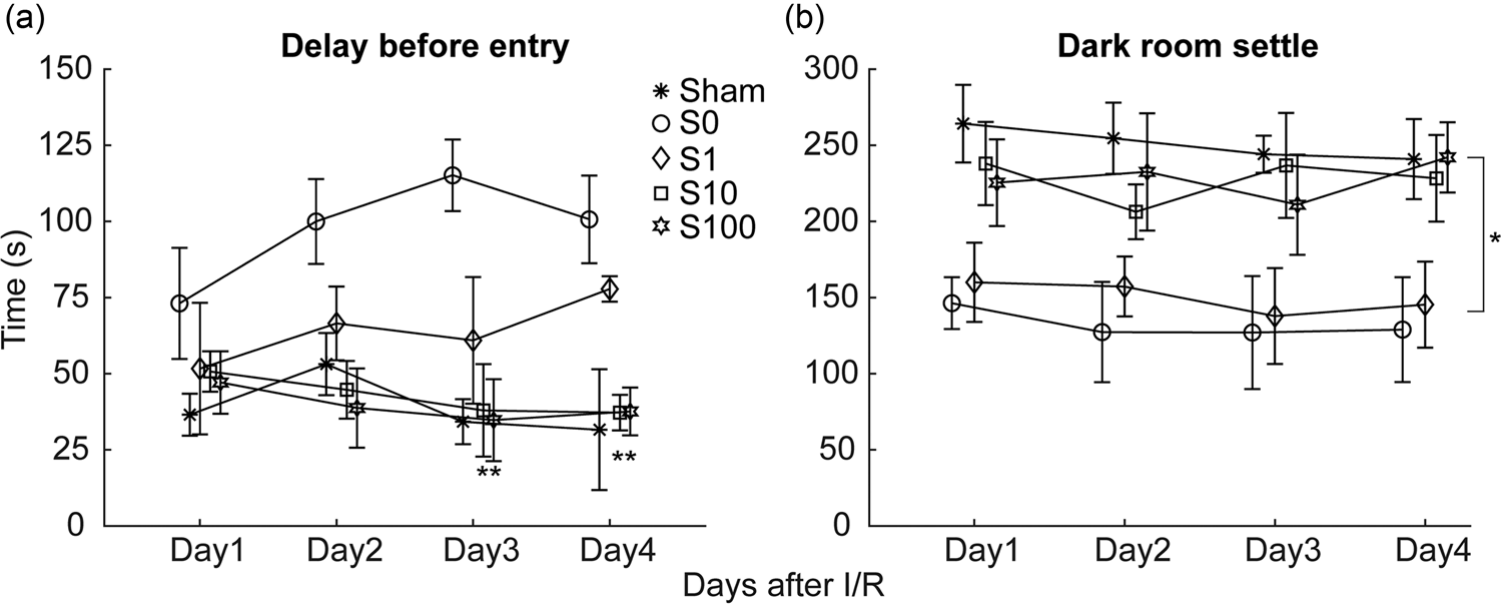
Saikosaponin A further ameliorated the learning defects induced by the I/R treatment. (a) The animals learned to stay in a lighted room to avoid electric shock in the darkroom. For 4 consecutive days, the model and S1 groups did not show an advance of learning progress. As comparison, the S10 and S100 groups have significant learning effects when compared with the model group. (b) Animals with learning ability spend less time in the darkroom. For 4 consecutive days, the model and S1 groups stayed longer than the Sham group. In contrast, the S10 and S100 groups have significant learning effects when compared with the model group. * *p* < 0.05, ** *p* < 0.01, comparing S10 and S100 in groups A and B with the control group.

The passive avoidance test is an important method to evaluate cognitive function in rats, which avoids the bias caused by motor defects. After MCAO, I/R-treated rats showed a lower lag period and a higher darkroom time when compared with the sham group rats. S10 and S100 pretreatment increased delay time (Figure 3a) and reduced darkroom time (Figure 3b) when compared with the sham group rats, with statistically significant difference between each group.

### 3.3 SSa reduces brain ischemia and reperfusion damage in I/R rats

**Figure 4:**
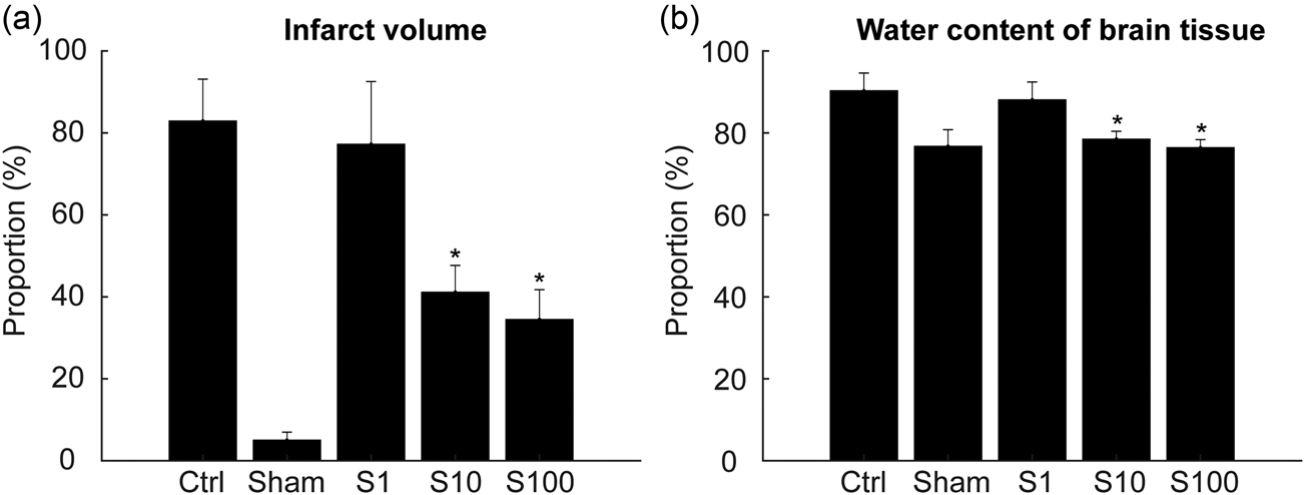
Saikosaponin A delivery reduced neurons damage induced by the I/R treatment. (a) Percentage of infarct volume after I/R treatment. Neuronal damage was significantly reduced in the S10 and S100 groups when compared with the model group. * *p* < 0.05, compared S10 and S100 with the model group. (b) The amount of water in brain tissue was significantly reduced in the S10 and S100 groups when compared with the model group. *p < 0.05, compared S10 and S100 with the model group.

The TTC method was used to assess ischemia levels to demonstrate the effect of SSa on ischemia injury. After MACO, the areas of cerebral infarction in rats treated with S10 and S100 were significantly reduced when compared with the model group rats (Figure 4a). After ischemia and reperfusion, cerebral tissue edema increased the brain water content (Figure 4b). When treated with different doses of SSa, the brain water content decreased significantly when compared with that of the sham group rats.

### 3.4 SSa reduces the increased LDH activities and inflammatory cytokine levels in the I/R rat serum

**Figure 5:**
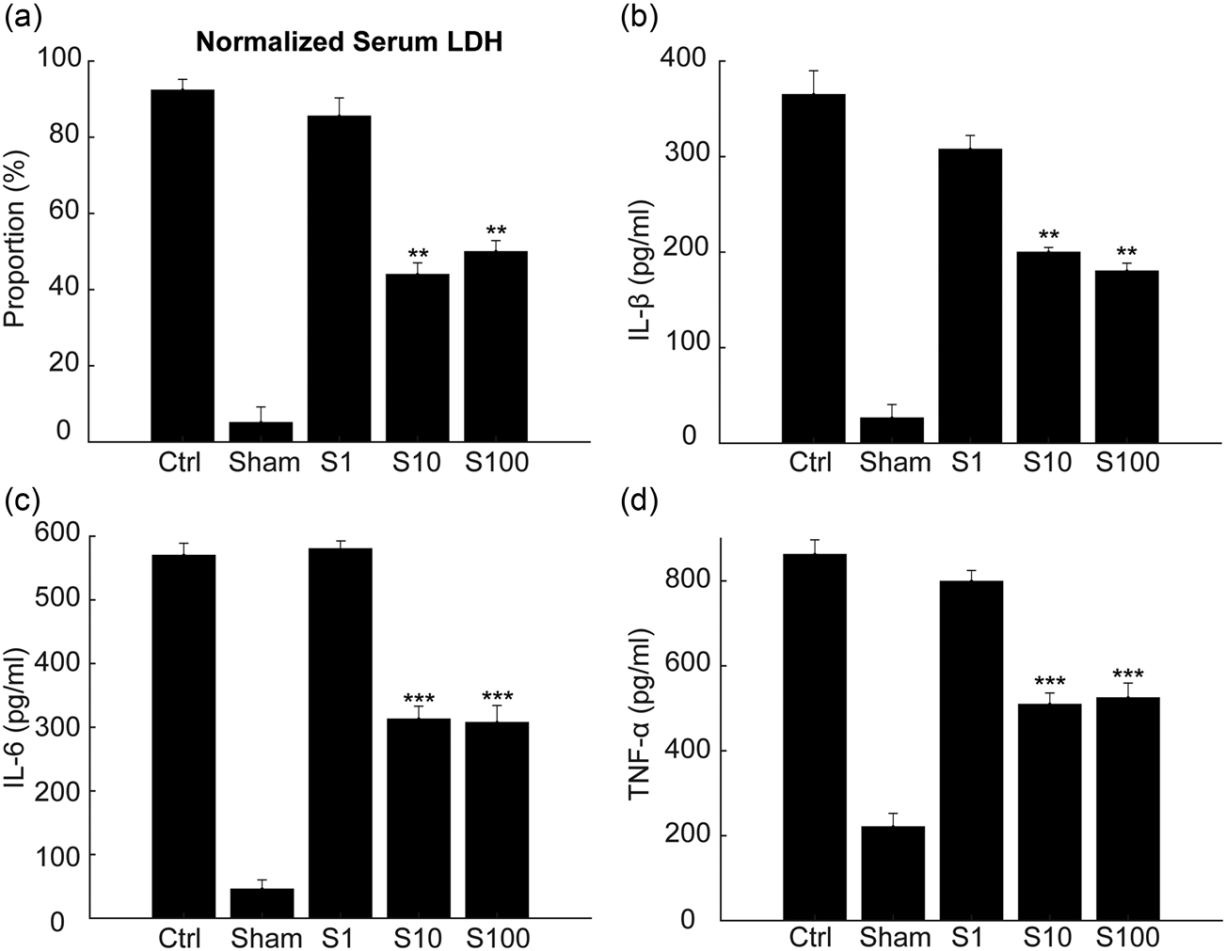
Tissue damage and inflammatory reaction were decreased by administration of Saikosaponin A. (a) 24 h after MCAO, serum LDH levels were significantly decreased in S10 and S100 groups when compared with the model. ** *p* < 0.01, compared S10 and S100 with the model group. (b–d) ELISA results showed that Saikosaponin A treatment attenuated the increase in serum cytokines. IL-1β (b), IL-6 (c), and TNF-α (d) were all decreased in S10 and S100 groups when compared with the model group. ** *p* < 0.01, ***p < 0.0001, when compared S10 and S100 with the model group.

When an inflammatory response occurs in the tissue, the levels of LDH and inflammatory cytokines in the serum increase accordingly. Rats treated with I/R lacked blood oxygen supply, causing the inflammatory response that further leads to apoptosis and necrosis. Therefore, we tested the levels of LDH and inflammatory cytokines in the serum to verify the protective effect of SSa. The results showed that SSa reduced IL-1β, IL-6, TNF-α, and LDH levels in the serum of MACO rats, indicating that SSa inhibited the inflammatory response in the I/R rats (Figure 5a–d).

### 3.5 SSa downregulates the protein expression levels of TLR4 and NF-κB in I/R rats’ brain

**Figure 6:**
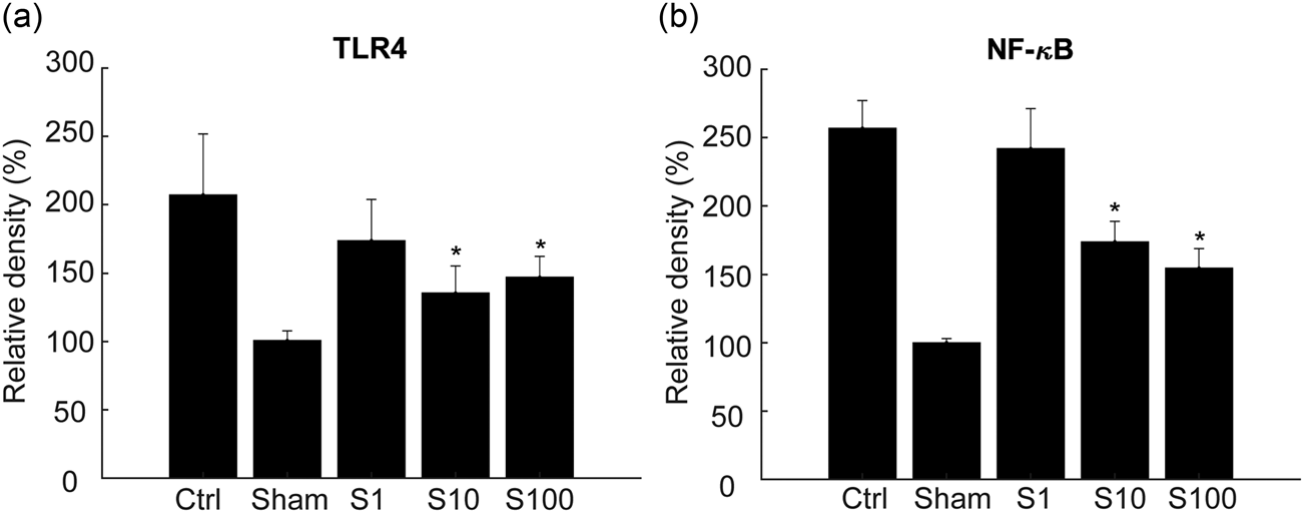
TLR4 and NF-κB expression were reduced by Saikosaponin A treatment. Group S10 and S100 treatment significantly decreased the expression of TLR4 (a) and nucleus NF-κB (b) when compared with the model group. * *p* < 0.05, compared S10 and S100 with the model group.

The expression levels of TLR4 and NF-κB in ischemic brain tissue increased at 24 h after cerebral ischemia, but SSa significantly downregulated the expression of TLR4 and NF-κB (Figures 6a and b) as indicated by the Western blot analysis. Furthermore, it was found that the SSa treatment (0.01%) significantly decreased the expression of TLR4 and nucleus NF-κB 4 days after stroke (supplementary Figure S1a and b).

### 3.6 SSa inhibits nuclear HMGB1 release

**Figure 7:**
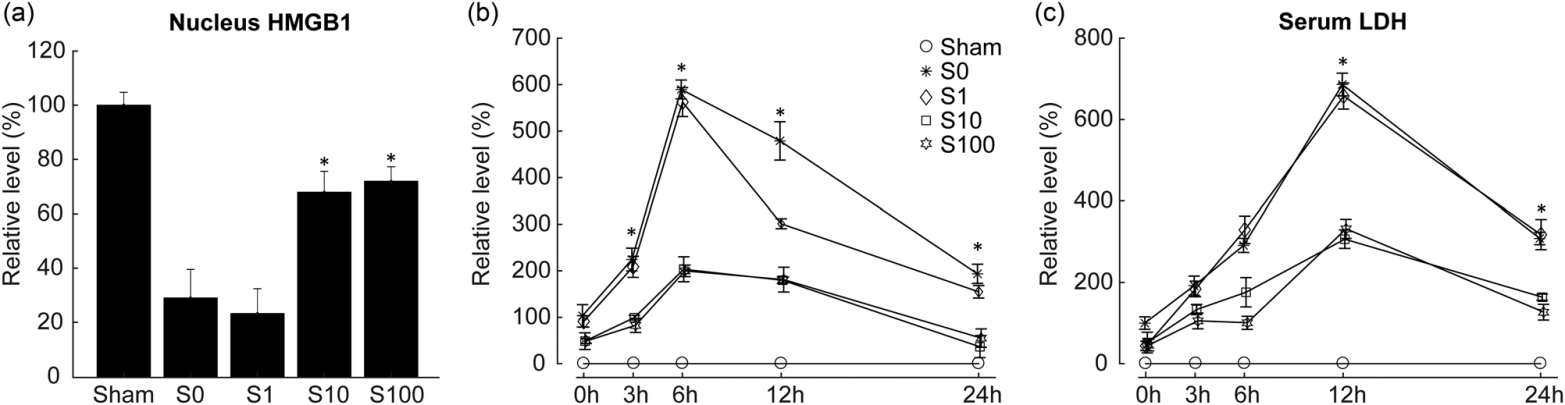
Saikosaponin A inhibited release of nucleus HMGB1, thus prevented the inflammatory effect. (a) Rats were sacrificed after MACO to detect the HMGB1 level. S10 and S100 groups restore the amount of HMGB1 as the Sham group, indicating that Saikosaponin A inhibited the release of HMGB1 from nucleus. (b and c) The serum levels of HMGB1 and LDH were detected. S10 and S100 groups reduced inflammation when compared with the model group, indicating that Saikosaponin A suppressed inflammation.

HMGB1 released from the nucleus leads to the activation of nuclear NF-κB, resulting in upregulation of proinflammatory cytokines and exacerbation of cellular oxidative stress. Our results indicated that SSa inhibited the release of HMGB1 from the nucleus of the MACO rat brain by Western blot (Figure 7a). In addition, SSa pretreatment reduced HMGB1 release into the serum during reperfusion (Figure 7Bb) and also reduced LDH release (Figure 7c), indicating attenuated nerve damage caused by brain I/R. All these results indicated that SSa reduced brain I/R-induced neuronal damage, likely by inhibiting HMGB1 release.

## 4 Discussion

Ischemic stroke is one of the most common neurological diseases leading to high mortality and disability and has not yet been effectively treated [1]. Therefore, it is necessary to explore novel treatment methods. Timely reperfusion can definitely prevent severe neurological deficits and death, but it may also exacerbate neuronal death and neurological deficits. A variety of pathophysiological mechanisms are related to these adverse reactions, such as intracellular Ca^2+^ elevation, energy metabolic failure, mitochondrial damage, and inflammatory response [4,20]. A growing body of evidence supports the view that inflammation is detrimental in I/R-induced damage. However, the exact mechanism is still unclear, with respect to how these inflammatory cytokines are regulated by physiological and pathological stimulating regulators.

HMGB-1, released from necrosis and dying cells in the ischemic brain, can trigger TLR4 activation [8]. Activated TLR4 undergoes a series of signal transductions, stimulating NF-κB nuclear translocation and then causing the release of inflammatory factors and I/R injury [21,22]. Therefore, therapeutic strategy targeting at HMGB-1/TLR4/NF-κB signaling may be a promising method to limit neuroinflammatory processes and alleviate stroke damage.

Acute neuroprotection after ischemia is very important, and the neuroprotective strategy aims to reduce ischemia-induced damage. Traditional Chinese Medicine has attracted much attention for its rich clinical experience in the cerebral I/R treatment [23,24]. The main bioactive compound isolated from the dried root of *R. bupleuri* is Saikosaponin, which has been shown to have many beneficial properties such as anti-inflammatory, antibacterial, antiviral, and immunomodulatory activities. SSa showed potent anti-inflammatory activity by inhibiting NF-κB activation, iNOS, COX-2, and proinflammatory cytokines [16]. Another study demonstrated that SSa treatment attenuated the destruction of the BBB and improved functional recovery after traumatic brain injury. The therapeutic effects were associated with SSa-induced inflammatory inhibition, likely through suppressing the MAPK signaling pathway [25]. Based on the above literature, whether SSa can alleviate cerebral I/R injury attracted our interest, and this study may provide a potentially novel agent for treating I/R injury. Our study used the rat MACO model to explore the role of SSa in inhibiting neuroinflammation and improving brain damage after ischemic stroke, as well as its potential pharmacological mechanisms.

Brain I/R injury can lead to cerebral edema and neurological deficits, accompanied by elevated serum LDH [26]. Our results showed that SSa attenuated the impaired motor and cognitive functions in the MACO rat (Figures 2 and 3), significantly reduced the infarct area (Figure 4a), and decreased the brain water content (Figure 4b). All these results indicated that SSa had significant neuroprotective effects on cerebral I/R damage.

Inflammation caused by tissue damage is a complex process of pathology and physiology involving inflammatory factors secreted by monocytes, macrophages, and microglia [27]. Inflammation has been verified to be involved in the pathogenesis of brain I/R damage. We further studied the effect of SSa on the inflammatory responses induced by I/R injury. The results showed that the SSa pretreatment inhibited the increased serum levels of IL-1β (Figure 5b), IL-6 (Figure 5c), and TNF-α (Figure 5d) as expected.

When cells are injured or near death, their membranes are damaged, and HMGB1 is released from these impaired cells. HMGB1 activates inflammatory response, leading to NF-κB transcription [28,29]. Western blot results showed that nuclear HMGB1 levels were reduced in the model group at 24 h after MCAO, and SSa reversed this response, indicating that SSa inhibited the release of HMGB1 from the nucleus. At the same time, SSa pretreatment decreased the serum HMGB1 level at 3, 6, 12, and 24 h after MACO (Figure 7b), which was also accompanied by delayed inhibition on LDH release by SSa, indicating the degree of neuroinjury (Figure 7c). Simultaneously, SSa pretreatment reduced serum HMGB1 levels at 3, 6, 12, and 24 h after MACO (Figure 7b), accompanied by a delay in SSa inhibition on LDH release as well, indicating the severity of nerve cell damage. All of these data confirmed our hypothesis that SSa might suppress inflammation by inhibiting HMGB1 release. Consistent with these results, brain I/R in the model group also increased the levels of TLR4 and nuclear NF-κB (p-P65), which were significantly extenuated by the SSa pretreatment, suggesting that the inhibition of NF-κB activation may be related to the function of SSa.

In the current study, we demonstrated that SSa exerted protective effect on cerebral I/R injury in a rat MCAO model. SSa pretreatment could significantly reduce brain damage, improve neurological function recovery, and decrease the water content in brain tissue. The SSa pretreatment significantly reduced the serum HMGB1 level and downregulated the contents of inflammatory cytokines. Furthermore, the SSa pretreatment could attenuate nucleus NF-κB increase, indicating that HMGB1 release was inhibited, and anti-inflammatory response might be involved in the neuroprotective effect of SSa on brain I/R injury.

In summary, this study has demonstrated the protective effects of SSa on cerebral I/R injury using a rat MACO model, and the effects of SSa are at least partially associated with its inhibition of HMGB1 release, attenuation of NF-κB activation, and suppression of subsequent activation of inflammatory responses.

## Abbreviations

BBB: blood–brain barrier
COX-2: cyclooxygenase-2
HMGB1: high-mobility group box 1
I/R: ischemia/reperfusion
ICA: internal carotid artery
IL-6: interleukin-6
IL-β: interleukin-1 beta
iNOS: inducible nitric oxide synthase
LDH: lactate dehydrogenase
MCAO: middle cerebral artery occlusion
NF-κB: nuclear factor-kappa B
SSa: Saikosaponin A
TLR2: Toll-like receptor 2
TLR4: Toll-like receptor 4
TNF-α: tumor necrosis factor-α
TTC: 2,3,5-triphenyltetrazolium chloride.
